# Navigating a Microplastic Sea: How the Pacific Cupped Oyster (*Magallana gigas*) Respond to Microplastic Pollution in Lagoons

**DOI:** 10.3390/toxics12060429

**Published:** 2024-06-13

**Authors:** Gianluca De Rinaldis, Paolo Pastorino, Tommaso Scirocco, Claudia Sacchetti, Serena Anselmi, Francesca Provenza, Monia Renzi, Antonietta Specchiulli

**Affiliations:** 1 National Research Council, Institute of Nanotechnology (NANOTEC), 73100 Lecce, Italy; gianluca.derinaldis@nanotec.cnr.it; 2 Istituto Zooprofilattico Sperimentale del Piemonte, Liguria e Valle d′Aosta, 10154 Torino, Italy; 3 National Research Council—Institute for Marine Biological Resources and Biotechnology (IRBIM), 71010 Lesina, Italy; tommaso.scirocco@cnr.it (T.S.); claudia.sacchetti@irbim.cnr.it (C.S.); mrenzi@units.it (M.R.); 4 Bioscience Research Center, Via Aurelia Vecchia 32, 58015 Orbetello, Italy; serena.anselmi@bsrc.it (S.A.); francesca.provenza@bsrc.it (F.P.); 5 Department of Life Science, University of Trieste, 34127 Trieste, Italy; 6 National Biodiversity Future Center (NBFC), 90133 Palermo, Italy

**Keywords:** coastal lagoons, biomarkers, HDPE, pacific cupped oyster, oxidative stress

## Abstract

Microplastic pollution poses an escalating concern, particularly in coastal lagoons rich in biodiversity. This study delved into the occurrence of microplastics (MPs) in *Magallana gigas* (formerly *Crassostrea gigas*) from the Orbetello and Varano coastal lagoons (Italy), also investigating the response of these filter-feeding organisms to various colors (P = pink; B = blue; W = white) of high-density polyethylene (HDPE) MP fragments. Oysters were exposed for 7 days under controlled conditions. Subsequently, the oysters underwent analysis for both MP presence and biochemical markers of oxidative stress. Diverse ingestion rates of HDPE were noted among oysters from the two lagoons, eliciting antioxidant responses and modifying baseline activity. The two-way ANOVA revealed the significant effects of treatment (control; HDPE_B; HDPE_P; HDPE_W), site, and the interaction between treatment and site on all biomarkers. Non-metric multidimensional scaling showed a divergent effect of HDPE color on biomarkers. Further investigation is warranted to elucidate the mechanisms underlying the influence of MP color, dose-dependent effects, and the long-term impacts of exposure. Comprehending these intricacies is imperative for devising effective strategies to mitigate plastic pollution and safeguard marine health.

## 1. Introduction

The pervasiveness of microplastic (MP) particles in marine ecosystems is a growing concern [1]. While research indicates potential adverse effects for particles sized between 0.1 μm and 5 mm [1,2], a comprehensive understanding of these impacts is still evolving [3,4]. Further investigations are essential to fully grasp the long-term consequences for ecosystem health, given their potential impact on biodiversity and the environment.

Beyond direct harm, MPs present a potential threat as vectors for contaminants and new pathogens, exacerbating the negative effects on both the environment and natural biota [5,6]. The emergence of these new challenges may pose significant risks to marine ecosystems and potentially human health.

Due to their small size, MP particles readily infiltrate the feeding strategies of numerous filter-feeding organisms, such as bivalves [7]. Consequently, these organisms are particularly susceptible to MP contamination [7]. Research has shown that exposing bivalves to MP particles leads to significant alterations in their antioxidant enzyme systems [8]. Initially, an increase in reactive oxygen species (ROS) concentration occurs in the tissues of the exposed organism, triggering the production of antioxidant biomarkers crucial for ROS detoxification pathways. These biomarkers include superoxide dismutase (SOD), glutathione S-transferase (GST), glutathione peroxidase (GPx), and malondialdehyde (MDA), an indicator of lipid peroxidation (LPO) [8].

Imagine a coastal lagoon: a sheltered, shallow body of water where freshwater mixes with the sea. Sand banks, reefs, or barrier islands create this unique, brackish environment teeming with life [9]. This is the essence of a coastal lagoon, a vibrant transitional environment shaped by the interplay of land and sea, rich in biodiversity and with a high productivity [9,10,11,12].

Despite their crucial role in providing ecosystem services, coastal lagoons face significant threats. Human activities such as aquaculture, agricultural runoff, and urban expansion contribute to their degradation [11,13]. However, plastic pollution is a new and growing concern.

Since the 1950s, our reliance on plastic has increased dramatically. Its low cost, durability, and resistance to degradation have led to its widespread use and, unfortunately, its release into the environment [6,14,15]. This plastic influx has significantly impacted marine ecosystems, and coastal lagoons are no exception [16,17,18]. Once vibrant biodiversity hotspots, lagoons are now becoming “hotspots of plastic accumulation” [10].

Oyster farming is a crucial economic activity in many lagoons, providing jobs and food for local communities. This is the case of two Italian lagoons: Orbetello in the North Tyrrhenian Sea and Varano in the South Adriatic Sea. These lagoons, renowned for their unique geomorphological features, are crucial transitional ecosystems rich in habitats and biodiversity. They also support intensive aquaculture and fishery activities [12,19,20]. Due to their contrasting morphologies and hydrological regimes, the lagoons experience anthropogenic pollution from shell farming activities to varying degrees [9].

Similarly, to other transitional environments, these two Italian lagoons face a growing threat: MP accumulation. Bivalves like oysters filter feed at the water’s surface, making them susceptible to ingesting MPs [15,21,22]. This is why they are valuable bioindicators, allowing the assessment of MP contamination levels by measuring their accumulation in oyster tissues [21,22,23]. While the risks of human consumption of these MP-accumulating oysters are still under investigation [24], this study, alongside that of Provenza et al. [25], aims to bridge that knowledge gap. Microplastic analyses were performed in the *Magallana gigas* (Thunberg, 1793), formerly *Crassostrea gigas*, collected from the two lagoon systems to define baseline levels of MPs and biochemical biomarkers. Moreover, the biochemical effects induced by the exposure of *M. gigas* to different colors of high-density polyethylene (HDPE) were determined. HDPE was chosen as the model for MPs due to its widespread use and resulting abundance in aquatic environments [25]. A variety of colors can be found in the aquatic environment, with pink, blue, and white being the most commonly observed in lagoon ecosystems [26,27]. In aquatic environments, the majority of plastic particles are filaments, with fragments being the second most prevalent shape [28]. It is important to note that the harmful effects of small plastic fragments on marine species are not yet fully understood, highlighting the need for further research in this area [29]. Therefore, fragment particles were chosen for this study.

## 2. Materials and Methods

### 2.1. Samples Collection and Study Area

**Figure 1:**
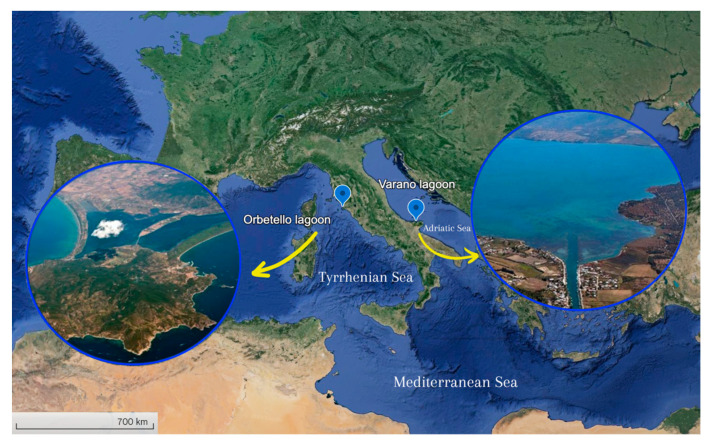
Orbetello (42°28′15.48″ Lat. N—11°11′52.24″Long. E) and Varano (41°53′38.75″ Lat. N—15°42′38.82″ Long. E) Lagoons (Italy) where specimens of *Magallana gigas* were collected.

In December 2019, 26 specimens of *M. gigas* were collected from two distinct Italian lagoons: Orbetello (n = 13) in the North Tyrrhenian Sea and Varano (n = 13) in the South Adriatic Sea (Figure 1). In partnership with local shellfish farmers, oyster specimens were collected from lagoon farms. Upon reaching the laboratory, they were placed in tanks filled with lagoon water and carefully cleaned using filtered seawater to eliminate any debris. Following this, the length of each specimen was measured using calipers (precision ± 0.1 mm).

The Orbetello Lagoon, located on the west coast of Tuscany, spans approximately 2300 ha with an average depth of 1 m. It has been extensively studied due to its exposure to both natural and human-induced pressures [30]. Eutrophication is a significant issue in this coastal lagoon, exacerbated by the coexistence of various submerged vegetation types, resulting in uneven biomass decomposition and phanerogam seed dispersal. The lagoon’s shallow brackish waters, limited circulation, low volume, and partial isolation from the sea hinder its ability to dilute nutrients and contaminants from urban, aquacultural, and agricultural sources. Sediment analysis has revealed elevated levels of trace elements like arsenic, cadmium, chromium, copper, mercury, nickel, lead, and zinc [30]. In contrast, the Varano Lagoon, situated on the northern coast of the Gargano Promontory in the Southern Adriatic Sea, covers 6500 ha with an average depth of 4 m. It is partially isolated by coastal barriers, with communication to the sea facilitated by channels influenced by tidal and wind dynamics. Salinity levels remain relatively stable, and the hydrological system includes freshwater inflows, a drainage pumping station, and various springs. The lagoon’s water balance involves a daily freshwater input of around 87,000 m^3^, sourced from urban and agricultural runoff, fish farming, and other activities. Limited tidal movement and restricted exchange with the coastal area result in a basin-wide average water renewal time of about 260 days. While mussel farming has historically been significant, fishing has become the primary economic activity in recent years [31].

Both lagoons face unique ecological challenges and anthropogenic impacts, highlighting the importance of ongoing research and management efforts to preserve these fragile ecosystems.

### 2.2. Laboratory Experimental Protocol

All oysters underwent a 15 day acclimation period to standard environmental conditions, including a salinity of 36‰, pH of 8.12, and temperature of 20 ± 1 °C, as outlined by Provenza et al. [25]. After acclimation, oysters from Orbetello and Varano were assigned to specific treatment groups, following a 2 (location) × 3 (color) × 3 (replicates) design, resulting in 18 treatment groups and one set of negative control triplicates. Each oyster was exposed to its allocated treatment in an individual glass jar under controlled conditions for 7 days, with three replicate jars utilized for each treatment group. Each jar contained 1 L of naturally filtered seawater (0.45 μm) and maintained consistent physicochemical parameters, mirroring those of the acclimation phase (salinity 36‰, pH 8.12, temperature 20 ± 1 °C). Each glass jar was continuously oxygenated during the experiment using an air pump (Newair1, Newa, Italy). Filtered seawater was examined for the presence of MPs (methodology detailed in Section 2.3), and no MP items were recorded.

Exposure involved a single concentration (0.05 g/L) of fragmented high-density polyethylene (HDPE) available in three colors: pink, blue, and white. This concentration was chosen based on prior experimental exposure studies conducted on marine mussels (*Mytilus galloprovincialis*) [25].

Oysters were exclusively assessed under fed conditions, with a daily introduction of a fish feed mixture into the water at a concentration of 30 mg/L [25]. The fish feed was pre-tested for the presence of MPs according to the methods described in Section 2.3, and no MPs were detected.

At the onset of the experiment (T_0_), MPs were introduced, and consistent water conditions were maintained in each container throughout the entire exposure duration. Parameters such as temperature, light/dark cycles, oxygen levels, and salinity were monitored daily as reported by Provenza et al. [25]. Each experimental group included a negative control consisting solely of filtered seawater. Regular mortality checks were carried out each day to determine the final mortality percentage for each group. At the conclusion of the exposure period (T_7_), biochemical biomarker analysis was conducted following the procedures outlined in Section 2.4. To minimize errors of both Type I and Type II, the experiment was structured based on a logical model [32] with a nested hierarchical arrangement. This design incorporated predetermined factors of variability selected at random.

The experiment investigated the impact of two primary factors:

- Oyster origin: Oysters were sourced from two distinct locations—Orbetello and Varano Lagoons. These locations were considered fixed effects within the study design;
- Microplastic color: Three different hues of high-density polyethylene (HDPE) were examined—pink (HDPE_P), blue (HDPE_B), and white (HDPE_W). Color served as another fixed effect.

### 2.3. Microplastics Determination

The determination of MPs was assessed in each individual (n = 6 oysters for both Varano and Orbetello) at the beginning of the experiment (T_0_) and after the HDPE exposure (T_7_) following the methodology outlined by Provenza et al. [25]. Briefly, oyster tissues were meticulously dissected to prevent any potential contamination and then assessed under a microscope (Nikon, mod. P-DSL32, Tokyo, Japan) connected with a camera (Nikon, mod. DSFi3). Following digestion with Creon (37 °C; TRIS-buffered pH) and filtration (pore 0.6 μm), particles underwent measurement and chemical analysis through a combination of microscopy and Fourier-transform infrared spectroscopy (μFT-IR). This analysis utilized a Thermo i-10 Nicolet MX infrared imaging microscope (Thermo Fisher Scientific, Waltham, MA, USA) equipped with a detector optimized for operation at room temperature across the spectral range of 7600–450 cm^−1^ and with liquid nitrogen within the range of 1800–650 cm^−1^. Plastic particles were tallied, their chemical composition was determined, and they were categorized using a database integrated into the Thermo Scientific™ OMNIC™ Picta™ software.

### 2.4. Biomarker Activity Evaluation

At the beginning (T_0_) and at the end (T_7_) of the experiment, biomarkers associated with oxidative stress were assessed in oysters from both lagoons. These biomarkers, comprising superoxide dismutase (SOD), glutathione peroxidase (GPx), glutathione S-transferase (GST), and lipid peroxidation (LPO), were assessed via chemical analysis. Specifically, the examination focused on the digestive glands, renowned for their heightened generation of reactive oxygen species (ROS) [33,34]. Adhering to the methodology delineated in Provenza et al. [25], the digestive glands were harvested at T_0_, weighed, swiftly frozen in liquid nitrogen, and subsequently subjected to analysis for the oxidative stress biomarkers (SOD, GPx, GST, LPO). Three analytical replicates were processed for each sample (n = 3 per HDPE color).

The analysis utilized the soluble protein fraction obtained from the harvested tissues. To extract proteins, a phosphate buffer (50 mM, pH 7.4) containing EDTA (2 mM) [35] was added to the digestive gland homogenates at a ratio of 1:4 (*w*/*v*). EDTA was included to bind metal ions that could disrupt protein extraction. The homogenates were then disrupted using an Ultra-Turrax homogenizer, followed by centrifugation at 12,000× *g* for 12 min at 4 °C. This step separated the soluble proteins (supernatant) from cellular debris (pellet). The resulting supernatant, containing the soluble protein fraction, was divided into 2 mL tubes and rapidly frozen in liquid nitrogen for storage. This protein fraction was subsequently utilized for determining protein content and enzyme activity assays.

The protein concentration in the extract was measured using the Lowry colorimetric method [36]. This method relies on a chemical reaction that generates a colored product proportionate to the protein amount. Essentially, the protein samples were reacted with sodium hydroxide, Folin–Ciocalteu reagent, and a copper sulfate mixture. The absorbance of the resulting solution was measured at 750 nm, and the protein concentration was calculated based on a standard curve. Typically, results are expressed in micrograms of protein per milliliter (μg/mL).

The SOD activity was assessed according to the protocol outlined by Gao et al. [37]. This method employs pyrogallol, a compound prone to spontaneous oxidation. SOD mitigates this process, and its activity correlates with the extent of inhibition observed. The assay is conducted at pH 8.2 and monitored at 420 nm. One unit (U/mL) of SOD activity represents the quantity of enzyme necessary to induce a 50% reduction in pyrogallol autoxidation.

GST was assessed utilizing the procedure detailed by Habig et al. [38]. GSTs facilitate the conjugation of the substrate 1-chloro-2,4-dinitrobenzene (CDNB) with glutathione. The progression of the reaction is tracked by the reduction in absorbance of CDNB at 340 nm over a period of 5 min. GST activity is quantified as the quantity of CDNB conjugated per microgram of protein per minute (nmol/(μg×min)), considering the extinction coefficient of CDNB.

GPx activity was evaluated following the methodology established by Badary et al. [39]. This approach gauges GPx activity by its capability to diminish hydrogen peroxide using NADPH as an electron donor. The decline in NADPH is monitored at 340 nm. GPx activity is expressed as the quantity of NADPH oxidized per milligram of protein per minute (nmol/(mg×min)), with consideration given to the protein content.

LPO was assessed using 10% of the isolated S9 protein fraction. The colorimetric reaction was carried out with 1% phosphoric acid (*v*/*v*) and 0.6% thiobarbituric acid (*w*/*v*), following previously established methods [25]. After heating and centrifugation in 1-butanol, the malondialdehyde (MDA) content was measured spectrophotometrically at wavelengths ranging from 535 to 520 nm (with a minimum resolution of 1 nm). The findings were expressed as U mg^−1^ of protein.

### 2.5. Microplastic Features

Fragments of HDPE were prepared for the study. Initially, certified HDPE materials underwent grinding to form fragments, followed by meticulous washing to eliminate any potential impurities that could affect the outcomes [25]. For a comprehensive explanation of the microplastic fragmentation process, refer to Provenza et al. [25]. Subsequently, the size of these microplastic particles, available in blue, pink, and white colors, was accurately determined using a stereomicroscope (Nikon P-DSL32) connected to a camera (Nikon DSFi3) and software (NS-Elements D.4.60). Calibration of the software was conducted to ensure precise size measurement. The dimensional ranges were the following (mean values; range): HDPE_B = 3031.8 μm (160.2–4896 μm); HDPE_W = 3214.5 μm (137.5–4889 μm); HDPE_P = 3189.2 μm (150–4757 μm). Finally, prior to the commencement of the experiment, μFT-IR spectroscopy was employed to analyze the chemical spectra (Figure S1 Supplementary Materials).

### 2.6. Statistical Analysis

To evaluate statistically significant differences in biochemical biomarkers among oysters exposed to MPs, a two-way ANOVA was conducted. Data normality was confirmed through the Shapiro–Wilk test, while homoscedasticity was verified using the Levene test. The Mann–Whitney U test was used to compare baseline levels of biochemical biomarkers at T_0_. The independent variables in the two-way ANOVA encompassed the site (Orbetello vs. Varano Lagoon), treatment group (control, HDPE_B, HDPE_P, HDPE_W), and their interaction (site × treatment). Subsequent post-hoc analysis utilizing Tukey’s multiple comparison test was employed to pinpoint specific distinctions between the control group and groups exposed to various HDPE MPs (HDPE_B, HDPE_P, and HDPE_W). To visually represent potential trends in biochemical responses (SOD, GPx, GST, and LPO) associated with different microplastic colors, non-metric multidimensional scaling (NMDS) was utilized; a resemblance matrix was obtained using the Bray–Curtis measure. Statistical significance was established at *p* < 0.05. Statistical analyses were performed using R software (version 3.5.2) from RStudio, Inc. (Boston, MA, USA).

## 3. Results

### 3.1. Oyster Microplastics: Natural Levels across Lagoons

Microplastic analysis was performed on oysters from both lagoons at the start of the experiment (T_0_). No MPs of the targeted types (HDPE_B, HDPE_P, and HDPE_W) were found in oysters from the Orbetello Lagoon (n = 6; size: 95 ± 3.2 mm), while in those from Varano Lagoon (n = 6; size: 75.5 ± 3 mm), one filament of polyethylene (PE) was detected in one individual.

### 3.2. Microplastic Abundance in Oysters after HDPE Exposure (T7)

**Figure 2:**
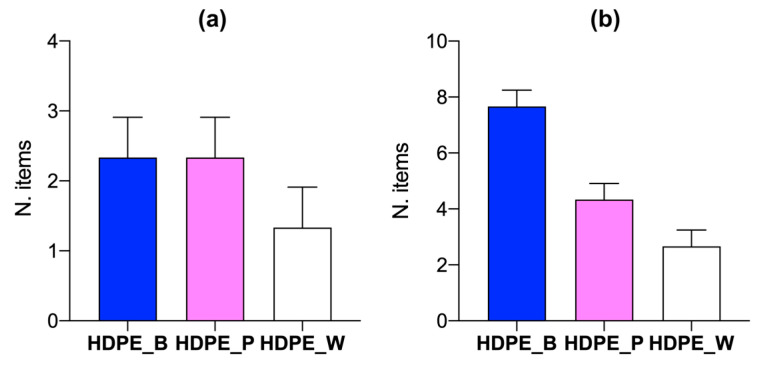
Microplastic (MP) abundances in oysters exposed to HDPE MPs (B = blue, P = pink, W = white), at the end of the experiment (T_7_), for fed oysters from Orbetello (**a**) and Varano (**b**).

Pacific oysters from Varano Lagoon ingested more MPs than those from Orbetello Lagoon (Figure 2). Oysters from Varano Lagoon preferentially ingested blue HDPE, with an average length of 759.6 ± 638.5 μm (range: 228.1–1985.4 μm), followed by pink HDPE (1188.3 ± 730.8 μm; range: 280.3–2336.7 μm) (Figure 2b). Oysters from Orbetello ingested both blue and pink MPs compared to white items (Figure 2a). Notably, from the data of all samples, Varano oysters tend to be smaller than those from Orbetello, with a size difference of approximately 15% observed in our study. To illustrate this point, Orbetello oysters have an average length of 88.2 ± 3.8 mm, whereas Varano oysters are on average 76.1 ± 3 mm. No mortality occurred during the exposure period in either the Varano or Orbetello oysters.

### 3.3. Biomarkers Values before (T0) HDPE Exposure

Baseline biomarker measurements before MP exposure revealed initial differences in antioxidant activity between oysters. Oysters from Orbetello Lagoon displayed higher, but not significant, SOD activity (2.37 ± 0.13 U/mg of proteins) compared to those from Varano Lagoon (2.15 ± 0.02 U/mg of proteins) (Mann–Whitney U test; U = 2; *p* = 0.4). This trend matched the pattern observed with the MDA level. Orbetello oysters exhibited significantly higher MDA levels (8.26 ± 0.79 μmol/min×mg) compared to the lower levels found in Varano oysters (3.49 ± 0.1 μmol/min×mg) (Mann–Whitney U test; U = 3; *p* = 0.02). Interestingly, oysters from Orbetello Lagoon displayed significantly lower GPx activity (34.69 ± 7.97 nmol/mg of protein) compared to the higher values (50.69 ± 0.27 nmol/mg of protein) of Varano (Mann–Whitney U test; U = 4; *p* = 0.036). The GST activity, however, presented similar values between lagoons: 0.13 ± 0.14 nmol/μg×min and 0.12 ± 0.02 nmol/μg×min in Orbetello and Varano, respectively.

### 3.4. Biochemical Biomarkers in Fed Oysters after (T7) HDPE Exposure

Table 1 presents the results of two-way ANOVA for treatment (control; HDPE_B; HDPE_P; HDPE_W), site, and the interaction of treatment × site on biochemical biomarkers. Analysis showed a significant effect (*p* < 0.05) of treatment, site, and the interaction of treatment × site on all biomarkers.

**Figure 3:**
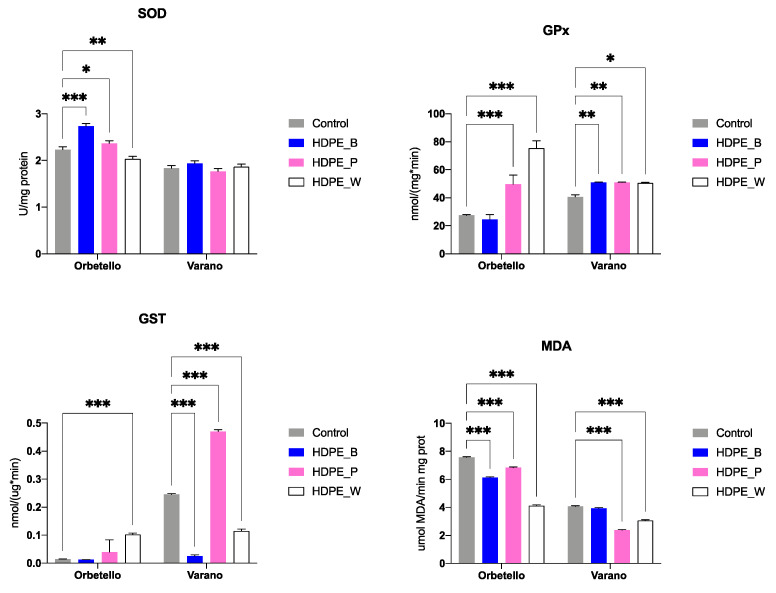
Biochemical biomarkers in oysters exposed to microplastics (HDPE): SOD = superoxide dismutase; GPx = glutathione peroxidase; GST = glutathione S-transferase; and MDA = malondialdehyde. Bars represent mean values ± standard deviation. Asterisks indicate significant differences between treatments according to Tukey’s multiple comparison test (* *p* ≤ 0.05, ** *p* ≤ 0.01, *** *p* ≤ 0.001). Treatments: control, HDPE_B (blue), HDPE_P (pink), HDPE_W (white).

The analysis of SOD activity significantly differed between control group and treated oysters (HDPE_B; HDPE_P; HDPE_W) from Orbetello (*p* < 0.05 for all comparisons) (Figure 3). Contrarily, levels of SOD were comparable to those of the control group in oysters from Varano (*p* > 0.05; Figure 3). GPx levels were significantly higher in oysters from Orbetello exposed to HDPE_P and HDPE_W. Significantly higher GPx levels were also recorded in oysters from Varano exposed to HDPE_B, HDPE_P, and HDPE_W (Figure 3). Significantly higher GST levels were observed in oysters exposed to HDPE_W from Orbetello and in oysters exposed to HDPE_P from Varano compared to the control group (*p* < 0.05); in contrast, significantly lower GST levels were observed in oysters from the Varano site exposed to HDPE_B and HDPE_W compared to the control (*p* < 0.05) (Figure 3). MDA showed significantly lower levels in oysters from Orbetello exposed to HDPE_B, HDPE_P, and HDPE_W compared to the control group (*p* < 0.05); the same trend was observed in oysters from Varano exposed to HDPE_B and HDPE_P (*p* < 0.05) (Figure 3).

### 3.5. Non-Metric Multidimensional Scaling (NMDS)

**Figure 4:**
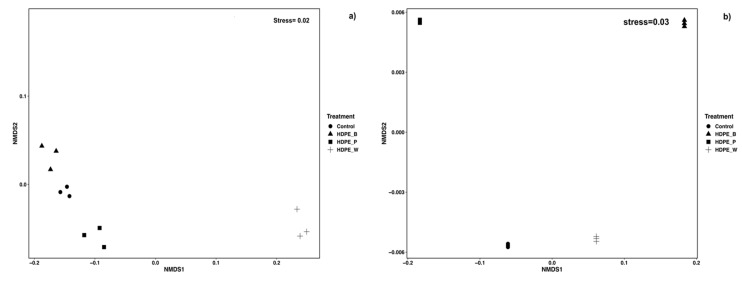
Non-metric multidimensional scaling (NMDS) of biochemical biomarkers (GST, SOD, GPx, MDA) in oysters exposed to microplastics (HDPE) from Orbetello (**a**) and Varano (**b**) lagoons. Points represent oysters exposed to control or different colored microplastics (HDPE_B = blue; HDPE_P = pink; HDPE_W = white). Closer points indicate more similar biochemical profiles.

To visualize potential differences in biochemical responses of oysters exposed to microplastics (MPs) compared to the control group, non-metric multidimensional scaling (NMDS) was applied to data from fed oysters. The analysis revealed a clear separation between the oysters exposed to MPs (HDPE_B; HDPE_P; HDPE_W) and the control group (Figure 4), indicating a different effect of HDPE color on biochemical biomarkers in oysters from both Orbetello (Figure 4a) and Varano (Figure 4b).

## 4. Discussion

This study investigated the occurrence of MPs in *Magallana gigas* from two Italian coastal lagoons: Orbetello, in the Tyrrhenian Sea, and Varano, in the Adriatic Sea. Then, the experiment examined how these filter-feeding organisms from these two lagoons respond to exposure to different colored HDPE MPs.

Microplastic contamination appears to be evident in Varano Lagoon based on environmental samples. Indeed, prior to the experiment, only one oyster from Varano Lagoon contained MP items. It is noteworthy that the PE item recovered from one specimen was of a different shape (filamentous) compared to the fragments of HDPE utilized in the study. This discovery may contradict previous research indicating widespread MPs in marine environments [1,6]. Two possible explanations for this disparity arise. Firstly, the relatively limited number of oysters examined might imply that researchers simply did not analyze enough oysters to detect MPs present. With a larger sample size, they might have detected MP items in more oysters, thus aligning the results with existing knowledge. Alternatively, it is conceivable that the waters in these lagoons are cleaner than other marine environments, resulting in a lower overall presence of MPs in the oysters themselves.

Polyethylene (PE) constituted the only MP chemical type detected in oysters collected from the Varano Lagoon. These observations underpin the rationale for selecting HDPE in our experimental investigation. Indeed, HDPE is a specific type of PE characterized by a higher degree of crystallinity and molecular weight compared to other forms of PE [40]. This results in a more tightly packed molecular structure and higher density commonly used in applications requiring high strength and durability, such as pipes, bottles for household and industrial chemicals, and containers for various liquids and powders [41]. Goldstein et al. [42] demonstrated that MP concentrations ranging from 10 μg/L to 0.1 mg/L are indicative of marine polluted areas. Similarly, Rosse and Loizeau [43] identified an environmentally significant range of MP concentrations from 10 μg/L to 0.5 mg/L, but recent trends show an escalation in production and corresponding levels observed in marine ecosystems [44]. In this study, we employed a high concentration of HDPE MPs (0.05 mg/L) to increase the likelihood of oysters ingesting them, a phenomenon documented previously in *M. galloprovincialis* [25].

Oysters were assessed to establish their initial levels (T_0_) of biochemical biomarkers. The presence of environmental contaminants and various abiotic factors prompts the activation of their defense mechanisms, leading to the regulation of antioxidant enzymes [33,34]. For this pathway, this study revealed significant differences in baseline antioxidant activity between the two lagoon populations. Notably, levels of SOD and GST did not show significant differences between ecosystems. However, oysters from Orbetello exhibited higher levels of MDA. Additionally, there were size differences among oysters collected from the two lagoons, influencing biomarker responses. Similar size-related variations in biomarker responses have been observed in the freshwater mussel *Dreissena bugensis* when exposed to environmental pollution [45]. The oxidative stress response may also vary due to differences in bioaccumulation, uptake, elimination, and leaching of chemical stressors, which are associated with the body size of the animals [46]. Seasonal changes can also affect biomarker responses. Ács et al. [45] noted a significant increase in lipid peroxidation (1.5–3.0-fold) and major DNA damage in mussels during colder months. Specifically, antioxidant enzyme activities in Dreissenidae species peak in late winter, coinciding with the early spawning stage of their reproductive cycle [47]. Seasonal variations in oxidative stress responses in mussels are also linked to changes in food availability [48]. Overall, these pre-existing variations likely influenced how each population responded to HDPE exposure. This highlights the importance of considering inherent physiological differences between organisms when assessing MPs’ impacts [4].

After 7 days of exposure, Varano oysters predominantly ingested blue HDPE MPs, likely due to their smaller size compared to the other types of MPs used. This phenomenon has been observed in other experimental studies as well.

For example, Graham et al. [49] exposed *Magallana gigas* specimens to fluorescent orange polystyrene MPs of known sizes (100, 250, and 500 μm), and found that the ingestion rate was higher for the smallest size class. However, various studies indicate that bivalves can consume larger particles. For example, blue mussels (*Mytilus edulis*) can ingest the early larval stages of sea lice (*Lepeoptheirus salmonis*), which average around 500 μm in size [50]. Additionally, a MP survey in the Dutch North Sea found that *M. gigas* contained large plastic particles, up to 5 mm in size.

Exposure for 7 days to HDPE MPs elicited diverse responses in the antioxidant defense systems of the oysters [51]. Orbetello oysters demonstrated heightened activity of SOD and GPx when exposed to certain colors of HDPE MPs. Conversely, Varano oysters exhibited a consistent increase in GPx activity regardless of the HDPE MP color.

Superoxide dismutase (SOD) activity was evident in the digestive gland of oysters from Orbetello, indicative of a demand for the conversion of surplus superoxide radicals (O_2_^•−^) into the less deleterious hydrogen peroxide (H_2_O_2_) to mitigate cellular oxidative damage following exposure to HDPE particles [52]. Consistent findings were also observed in *Mytilus edulis* exposed to polystyrene microplastics (PS-MPs) for 14 days at 32 μg L^−1^ [53], in the clam *Scrobicularia plana* exposed to PS-MPs at 1 mg L^−1^ [54], and in *M. gigas* exposed to irregular-shaped PE and PET MPs [52].

Glutathione peroxidase (GPx), responsible for eliminating H_2_O_2_, exhibited generally heightened activity. Indeed, a significant fluctuation in GPx levels was influenced by treatment and sampling locations, as evidenced by the results of the two-way ANOVA. This variability in biochemical response has been previously documented [55]. The considerable diversity found in the existing literature may stem from disparate experimental designs, particularly variations in exposure duration and microplastic concentrations [55]. Nonetheless, GPx appears highly responsive to MP exposure and has been proposed as a pivotal biomarker for evaluating MP-induced toxicity [55].

The levels of GST exhibited a comparable pattern, demonstrating either an increase or decrease in activity depending on the site and type of treatment. GST plays a crucial role in phase II detoxification [33]. Consistent with the variability observed in GPx levels, the literature also reports considerable variability in GST response [55]. For instance, heightened GST activity was observed in *M. galloprovincialis* exposed to leachates from plastic and tire rubber [56]. In contrast, the digestive gland of *M. galloprovincialis* exposed to chrysene-sorbed polystyrene MPs exhibited alterations in GST activity [57].

MDA, a resultant of lipid peroxidation, is commonly employed to assess oxidative stress. Its analysis in the digestive gland of mussels has been rigorously validated to monitor the progression of the ROS-induced impacts of micro/nanoparticles [57]. Notably, levels of MDA decreased in both lagoons following HDPE exposure. This apparently contradictory response hints at a potential compensatory mechanism in oysters to alleviate oxidative stress induced by MP ingestion [51].

The findings of this study highlight the intricate nature of MPs’ effects on marine organisms [57,58,59]. Factors such as the color of MPs and inherent physiological differences can influence responses [51]. While certain antioxidant enzyme activities increased in oysters, suggesting a potential compensatory mechanism to mitigate oxidative stress, as evidenced by decreased lipid peroxidation, further research is necessary to explore several key areas [33]. Firstly, the mechanisms by which the color of MPs influences ingestion and biochemical responses remain unclear. Future studies could delve into potential color-related interactions with the oysters’ feeding behavior or physiology, possibly through pigment analysis of ingested MPs [53]. Secondly, the experiment only utilized one concentration of MPs, limiting the understanding of dose-dependent effects.

The observed influence of the color of HDPE MPs on oxidative stress biomarkers in oysters, even under controlled conditions where all physicochemical water parameters except color were constant, suggests that color-specific factors are at play. Several hypotheses can be proposed to explain this phenomenon. One possibility is that the different dyes used to color the MPs have distinct chemical compositions, leading to varying levels of toxicity when these chemicals leach into the aquatic environment [60]. These chemicals may interact differently with the biological systems of the oysters, causing different levels of oxidative stress [61]. Another potential factor is the differential absorption of chemicals by MPs of different colors. Some colors may attract and retain more toxic substances, which could then be ingested by oysters, leading to increased oxidative stress [60]. Furthermore, the reactivity of the pigments to light could play a significant role. Certain colors might promote the generation of ROS under light exposure, thereby increasing oxidative stress in oysters [62].

Additionally, the surface properties of the MPs, which could be altered by the dyeing process, might affect how these particles are ingested and processed by the oysters [63]. Differences in surface texture or chemistry could influence the interaction between the MPs and the oysters’ digestive systems, potentially impacting the level of oxidative stress [64]. The potential for some pigments to possess photocatalytic properties should also be considered [65]. These properties could lead to the production of ROS upon light exposure, further contributing to oxidative stress in oysters. Finally, the bioavailability of the MPs could be affected by their color, with certain colors potentially being more easily ingested and assimilated by the bivalves [66]. This variation in ingestion and assimilation could lead to differing levels of oxidative stress, highlighting the complex interplay between the physical and chemical properties of the MPs and the biological responses of the oysters.

Examining a broader spectrum of concentrations would offer a more comprehensive understanding of how MPs impact antioxidant enzyme activity, potential bioaccumulation, and long-term health implications [18]. Furthermore, conducting long-term experiments is crucial to evaluate chronic effects such as potential bioaccumulation of MPs in oysters and the potential implications for human consumers who rely on them as a food source [24]. This research provides valuable insights into the potential ecological risks posed by MP pollution in coastal lagoon ecosystems. By comprehending these complexities and conducting further research along the suggested lines, we can develop more effective strategies to mitigate plastic pollution and safeguard the health of marine organisms.

## 5. Conclusions

This study investigated MP occurrence in *M. gigas* from Orbetello and Varano coastal lagoons in Italy, also assessing the response of these filter-feeding organisms to different colored HDPE MPs. Laboratory exposure revealed varied HDPE ingestion rates between lagoons, likely influenced by HDPE color and oyster size.

The study also explored antioxidant enzyme responses to MP exposure, revealing significant differences in baseline activity between lagoons and diverse responses to HDPE MP exposure, underscoring the complex effects of MPs on marine organisms. Further research is needed to elucidate the mechanisms underlying color-dependent ingestion and biochemical responses, explore dose-dependent effects, and assess long-term implications for ecosystem health and human consumption.

## Figures and Tables

**Table 1:** Results of two-way ANOVA of site (Orbetello; Varano), treatment (HDPE_P = pink high-density polyethylene; HDPE_B = blue high-density polyethylene; HDPE_W = white high-density polyethylene), and interaction (site × treatment) on biochemical biomarkers (GST = glutathione S-transferase; SOD = superoxide dismutase; GPx = glutathione peroxidase; MDA = malondialdehyde) in fed oysters. Degrees of freedom (dfn = numerator, dfd = denominator) and F statistics (F) are provided. Asterisks denote significant differences: *** *p* < 0.001; ** *p* < 0.01.

Biomarker	F Site	Site	F Treatment	Treatment	F Site–Treatment Interaction	F Site–Treatment Interaction
	(dfn, dfd)	(F-Value)	(dfn, dfd)	(F-Value)	(dfn, dfd)	(F-Value)
SOD	(1;16)	435.1 ***	(3;16)	49.46 ***	(3;16)	33.13 ***
GST	(1;16)	684 ***	(3;16)	220.1 ***	(3;16)	234.5 ***
GPx	(1;16)	9.17 **	(3;16)	94.18 ***	(3;16)	65.12 ***
MDA	(1;16)	1419 ***	(3;16)	1537 ***	(3;16)	994.8 ***

## Data Availability

The data presented in this study are available upon request from the corresponding author.

## Supplementary Materials

The following supporting information can be downloaded at: https://www.mdpi.com/article/10.3390/toxics12060429/s1, Figure S1: Spectra of high-density polyethylene (HDPE) particles (a: pink; b: blue; c: white) used in the present study.
