# Supplementary material for: Navigating a Microplastic Sea: How the Pacific Cupped Oyster (Magallana gigas) Respond to Microplastic Pollution in Lagoons
Source: Toxics. 2024 Jun 13;12(6):429. doi: 10.3390/toxics12060429 (PMC11209222; doi:10.3390/toxics12060429)
Supplement: Supplementary file 1 [file toxics-12-00429-s001.zip › toxics-3004468-supplementary.pdf]

Figure S1. Spectra of high-density polyethylene (HDPE) particles (a: pink; b: blue; c: white) used in the present study.

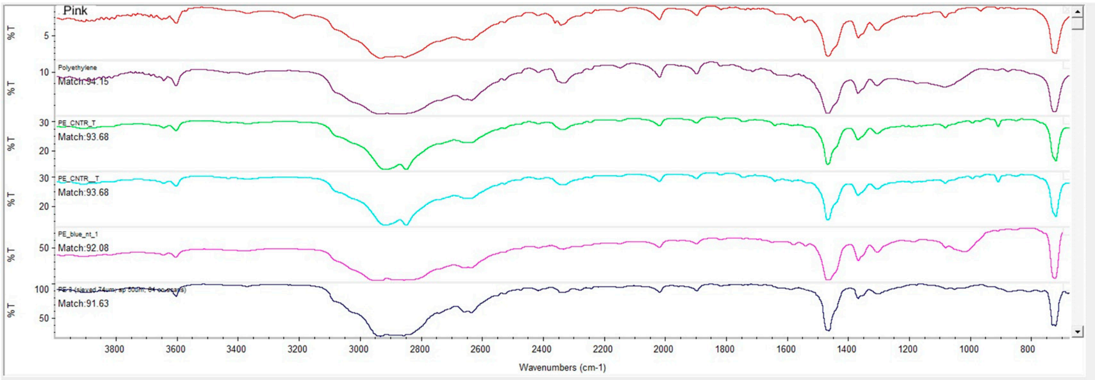

	Index	Match	Compound Name	Library Name
1	2	94.15	Polyethylene	Library di Riferimento BsRC
2	1	93.68	PE CNTR T	PE CNTR T
3	4	93.68	PE CNTR T	PE CNTR Completo
4	3	92.08	PE blue nt 1	Prove di Recupero 2020.01
5	18	91.63	PE 3 (sieved 74um; ap 50um; 64 co-scans)	Microplastics Reflection
6	1	91.53	PE white nt 1	Prove di Recupero 2020.01
7	20	90.82	PE 1 (sieved 74um; ap 50um; 64 co-scans)	Microplastics Reflection
8	12	90.81	PE 9 (sieved 74um; ap 50um; 64 co-scans)	Microplastics Reflection
9	13	90.25	PE 8 (sieved 74um; ap 50um; 64 co-scans)	Microplastics Reflection
10	21	90.17	PE (sieved 74um; ap 50um; 64 co-scans, 10 spectra AVERAGE)	Microplastics Reflection

a)

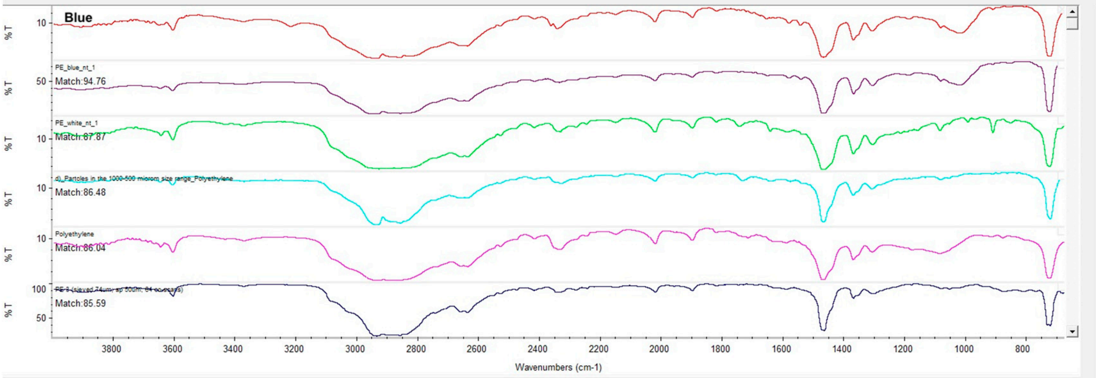

	Index	Match	Compound Name	Library Name
1	3	94.76	PE blue nt 1	Prove di Recupero 2020.01
2	1	87.87	PE white nt 1	Prove di Recupero 2020.01
3	6	86.48	d) Partcles in the 1000-500 microm size range Polyethylene	Plastiche Recupero
4	2	86.04	Polyethylene	Library di Riferimento BsRC
5	18	85.59	PE 3 (sieved 74um; ap 50um; 64 co-scans)	Microplastics Reflection
6	12	83.94	PE 9 (sieved 74um; ap 50um; 64 co-scans)	Microplastics Reflection
7	735	82.23	Linear low density polyethylene	HR Nicolet Sampler Library
8	13	82.09	PE 8 (sieved 74um; ap 50um; 64 co-scans)	Microplastics Reflection
9	20	80.93	PE 1 (sieved 74um; ap 50um; 64 co-scans)	Microplastics Reflection
10	21	79.92	PE (sieved 74um; ap 50um; 64 co-scans, 10 spectra AVERAGE)	Microplastics Reflection

b)

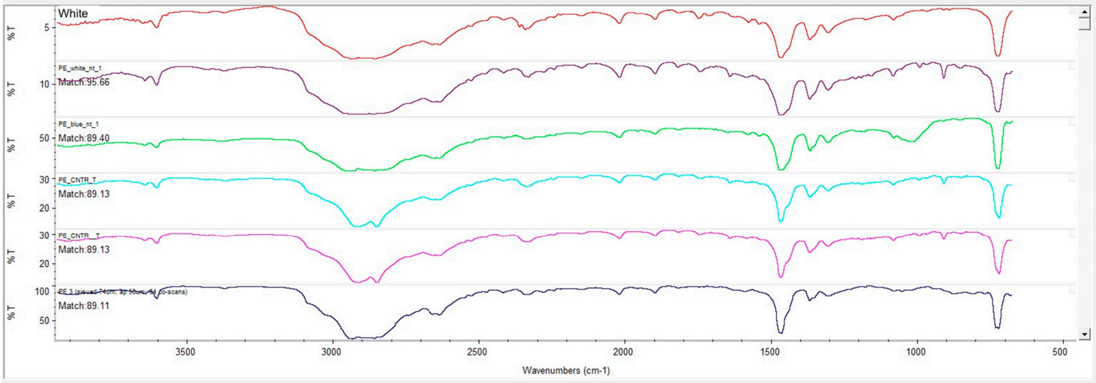

	Index	Match	Compound Name	Library Name
1	1	95.66	PE white nt 1	Prove di Recupero 2020.01
2	3	89.40	PE blue nt 1	Prove di Recupero 2020.01
3	1	89.13	PE CNTR T	PE CNTR T
4	4	89.13	PE CNTR T	PE CNTR Completo
5	18	89.11	PE 3 (sieved 74um; ap 50um; 64 co-scans)	Microplastics Reflection
6	735	88.47	Linear low density polyethylene	HR Nicolet Sampler Library
7	2	88.25	Polyethylene	Library di Riferimento BsRC
8	12	87.83	PE 9 (sieved 74um; ap 50um; 64 co-scans)	Microplastics Reflection
9	13	87.56	PE 8 (sieved 74um; ap 50um; 64 co-scans)	Microplastics Reflection
10	736	86.47	Polyethylene Marlex catalyst	HR Nicolet Sampler Library

c)
